# Supplementary material for: Cytoplasmic HIF-2α as tissue biomarker to identify metastatic sympathetic paraganglioma
Source: Sci Rep. 2023 Jul 18;13:11588. doi: 10.1038/s41598-023-38606-8 (PMC10354100; doi:10.1038/s41598-023-38606-8)
Supplement: Supplementary file 1 — Supplementary Information. [file 41598_2023_38606_MOESM1_ESM.pdf]

Cytoplasmic HIF-2α as tissue biomarker to identify metastatic sympathetic paraganglioma

Sinan Karakaya[1,2,3*], Lisa Gunnesson[4,5*], Erik Elias[4,5], Paula Martos Salvo[1,2,3], Mercedes Robledo[6], Ola Nilsson[7], Bo Wängberg[4,5], Frida Abel[8], Sven Påhlman[3,9], Andreas Muth[4,5†] and Sofie Mohlin[1,2,3†]

[1] Division of Pediatrics, Department of Clinical Sciences, Lund University, Lund, Sweden.

[2] Lund Stem Cell Center, Lund University, Lund, Sweden.

[3] Lund University Cancer Center, Lund University, Lund, Sweden.

[4] Department of Surgery, Sahlgrenska University Hospital, Gothenburg, Sweden.

[5] Department of Surgery, Institute of Clinical Sciences, Sahlgrenska Academy, University of Gothenburg, Gothenburg, Sweden.

[6] Hereditary Endocrine Cancer Group, Spanish National Cancer Research Centre (CNIO), 28029 Madrid, Spain.

[7] Department of Laboratory Medicine, Institute of Biomedicine, Sahlgrenska Academy, University of Gothenburg, Gothenburg, Sweden.

[8] Department of Clinical Genetics and Genomics, Sahlgrenska University Hospital, Gothenburg, Sweden.

[9] Translational Cancer Research, Department of Laboratory Medicine, Lund University, Lund, Sweden.

[*] *Shared first*

[†] *Shared last*

Correspondence to: Sofie Mohlin. Email: sofie.mohlin@med.lu.se; Phone: +46 – 462226418. Postal: Sölvegatan 19, BMC B11, Lund University, 223 84 Lund, Sweden.

Supplementary material

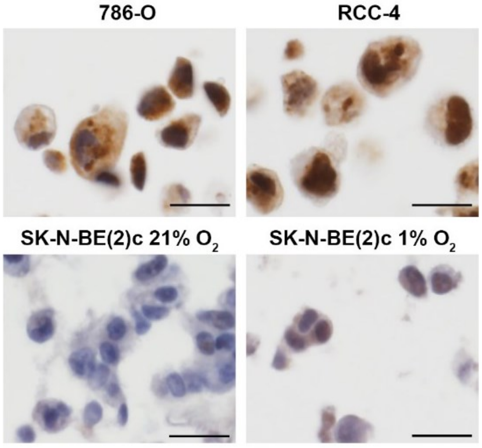

Fig. S1. *HIF-2α expression in renal cell carcinoma and neuroblastoma cell lines.* The specificity of HIF-2α antibody was tested by staining VHL-deleted renal cell carcinoma cells, 768-O (top-left) and RCC-4 (top-right) cultured under normoxia as well as neuroblastoma cell line SK-N-BE(2)c cultured under both normoxic (bottom-left) and hypoxic conditions (bottom-right).

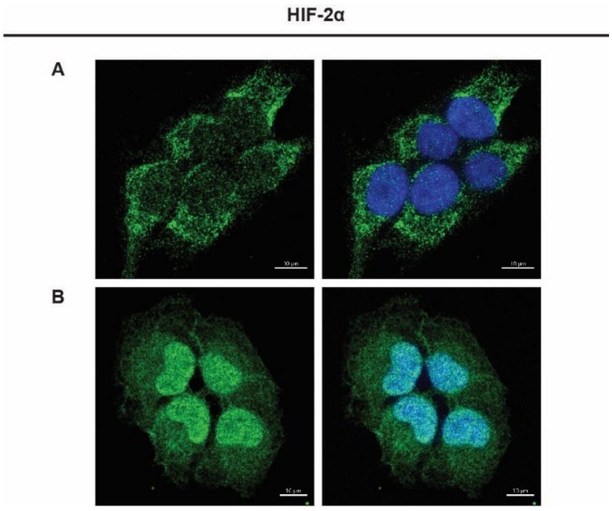

Fig. S2. *PPGL cell line hPheo1 express cytoplasmic and nuclear HIF-2α.* Cytoplasmic expression of HIF-2α was detected in hPheo1 cells by using two different antibodies from Abcam (ab207607) and Bethyl Labs (A700-003). Scale bar represents 10µm.

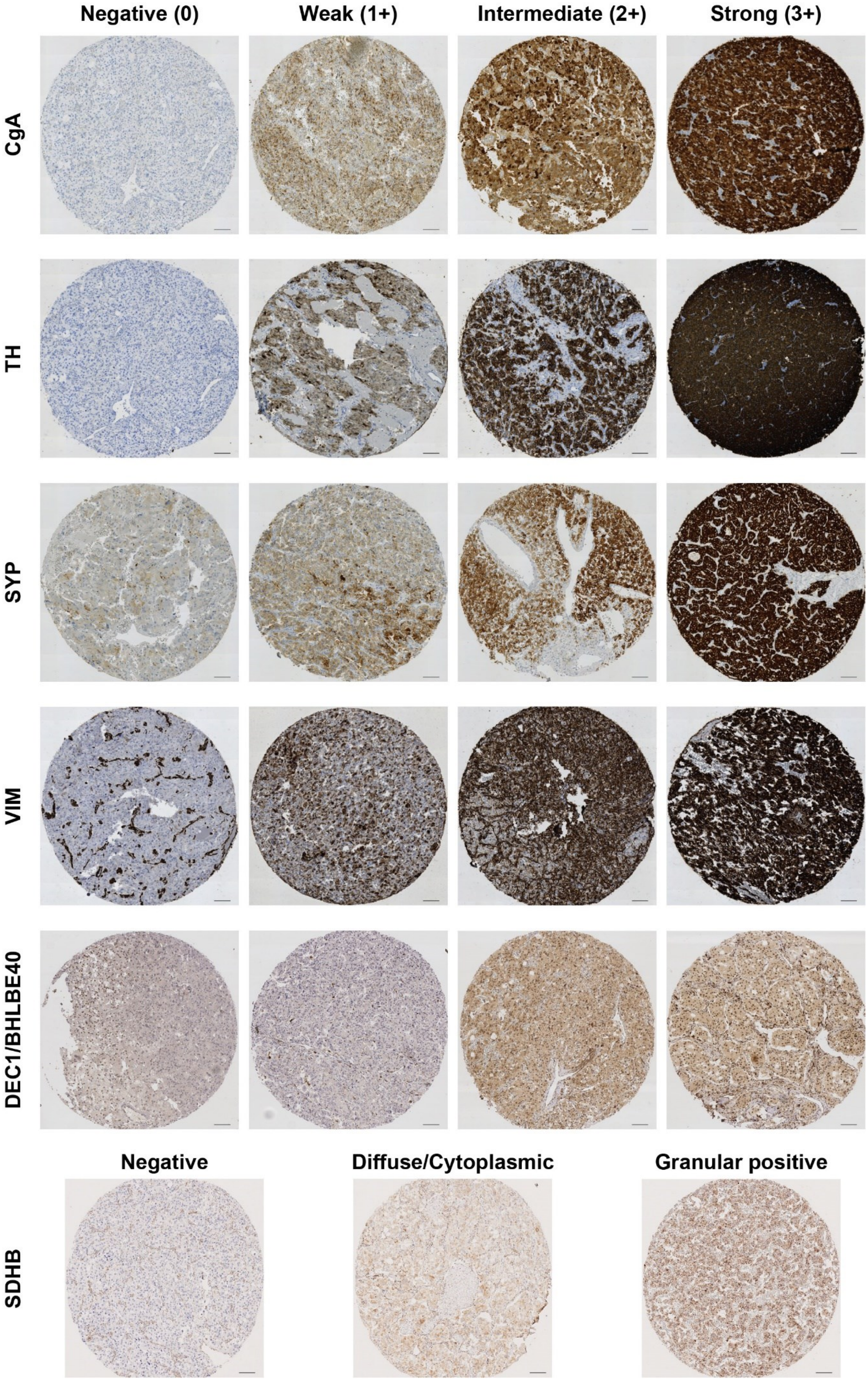

Negative (0)
Weak (1+)
Intermediate (2+)
Strong (3+)
CgA
TH
SYP
VIM
DEC1/BHLBE40
Negative
Diffuse/Cytoplasmic
Granular positive
SDHB

Fig. S3. PPGLs express clinically relevant markers. Representative images from tumor cores scored as negative (0), weak (1+), intermediate (2+) or strong (3+) for PPGL diagnostic markers CgA, TH, SYP, Vimentin, prognostic marker SDHB as well as HIF-2 downstream marker DEC1/BHLHE40. Scale bar represents 100μm.

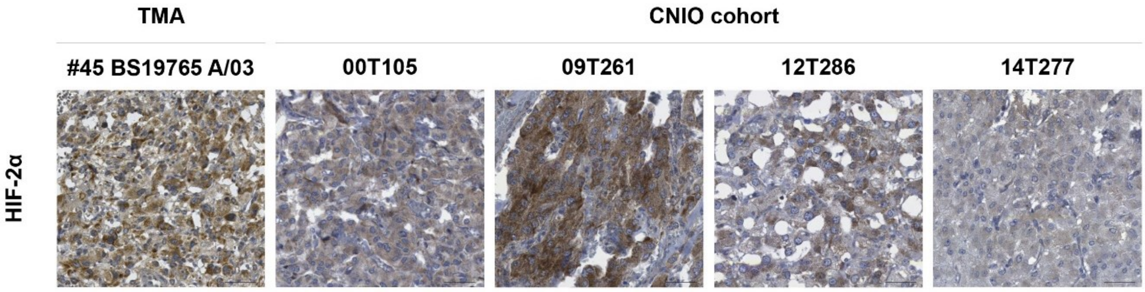

Fig. S4. *EPAS1 mutated tumors express cytoplasmic HIF-2α.* Images from tumor cores from five individual patients with mutations in the *EPAS1* gene from the TMA and CNIO cohort (Monteagudo *et al.* 2021) stained for HIF-2α. Scale bar represents 50μm.

Table S1. *Cohen's kappa coefficients for manual and QuPath scores of selected markers*

Marker	Weighted Kappa
HIF-2α-C	0,722
CgA	0,795
TH	0,909
SYP	0,775
VIM	0,937

Kappa < 0: No agreement
Kappa between 0.00 and 0.20: Slight agreement
Kappa between 0.21 and 0.40: Fair agreement
Kappa between 0.41 and 0.60: Moderate agreement
Kappa between 0.61 and 0.80: Substantial agreement
Kappa between 0.81 and 1.00: Almost perfect agreement.

Table S2. *Mutational information of PPGL patient cohort.*

Gene	Mutation	Diagnosis	Status
SDHx	SDHB: c.725G>A, p.R242H	2 PCCs	1 Somatic, 1 Germline
		1 sPGL	Germline
	SDHB: c.G600A, p.W200X	1 PCC	Germline
	SDHB: c.418G>T, p.V140F	1 sPGL	Germline
	SDHB: c.688C>T, p.R230C	1 HN-PGL	Germline
	SDHB: No data on specific mutation	1 sPGL	Germline
		1 HN-PGL	Germline
	SDHA: No data on specific mutation	1 PCC	Unknown
	SDHx: Negative SDHB-IHC	2 sPGLs	Unknown
		2 HN-PGLs	Unknown
RET	RET: c.2753T>C, p.M918T	3 PCCs	1 somatic, 2 Germline
	RET: c.1902C>G, p.C634W	1 PCC	Somatic
	RET: c.1900T>G, p.C634G	5 PCCs	Germline
	RET: c.1900T>C, p.C634R	2 PCCs	Germline
	RET: c.1826G>A, p.C609Y	1 PCC	Germline
	RET: c.634C>A	1 PCC	Germline
	RET: No data on specific mutation	4 PCCs	Germline
VHL	VHL: c.250G>C, p.V84L	1 PCC	Somatic
	VHL: c.301C>A	1 PCC	Germline
	VHL: c.392A>G, p.N131S	1 PCC	Somatic
	VHL: c.460C>A, p.P154T	1 PCC	Germline
	VHL: 505delTACCC	1 PCC	Germline
	VHL: deletion ex1	1 PCC	Somatic
NF1	NF1: c.7612del, p.M2538Wfs*11	1 PCC	Somatic
	NF1: No data on specific mutation	7 PCCs	6 Genomic, 1 Unknown
HRAS	HRAS: c.182A>G, p.Q61R	1 PCC	Unknown
	HRAS: c.35G>A, p.G12D	1 PCC	Somatic
EPAS1	EPAS1: c.1589C>T, p.A530V	1 sPGL	Somatic

Table S3. *CgA, TH, SYP, VIM, DEC1/BHLBE40 and SDHB staining characteristics of the cohort.*

Marker	Diagnosis/Intensity	Negative (%)	Weak (%)	Intermediate (%)	Strong (%)	Positive (%)	# of Tumors (%)
**CgA**	*sPGL*	3 (25)	4 (33)	4 (33)	1 (8)	9 (75)	12 (100)
	HN-PGL	6 (46)	5 (38)	2 (16)	0 (0)	7 (54)	13 (100)
	PCC	1 (1)	11 (9)	25 (21)	83 (69)	119 (99)	120 (100)
**TH**	*sPGL*	6 (50)	0 (0)	2 (17)	4 (33)	6 (50)	12 (100)
	HN-PGL	13 (100)	0 (0)	0 (0)	0 (0)	0 (0)	13 (100)
	PCC	3 (2)	9 (8)	13 (11)	95 (79)	117 (98)	120 (100)
**SYP**	*sPGL*	0 (0)	3 (25)	5 (42)	4 (33)	12 (100)	12 (100)
	HN-PGL	0 (0)	0 (0)	1 (8)	12 (92)	13 (100)	13 (100)
	PCC	1 (1)	6 (5)	14 (12)	99 (82)	119 (99)	120 (100)
**VIM**	*sPGL*	3 (25)	1 (8)	3 (25)	5 (42)	9 (75)	12 (100)
	HN-PGL	0 (0)	0 (0)	4 (31)	9 (69)	13 (100)	13 (100)
	PCC	13 (11)	13 (11)	23 (19)	71 (59)	107 (89)	120 (100)
**DEC1/BHLBE40**	*sPGL*	3 (25)	2 (17)	0 (0)	7 (58)	9 (75)	12 (100)
	HN-PGL	0 (0)	1 (8)	0 (0)	12 (92)	13 (100)	13 (100)
	PCC	28 (23)	32 (27)	13 (11)	47 (39)	92 (77)	120 (100)
**SDHB**	**Diagnosis/Intensity**	**Negative (%)**	**Diffuse/cytoplasmic (%)**		**Granular positive (%)**	**Positive (%)**	**# of Tumors (%)**
	sPGL	7 (54)	0 (0)		6 (46)	6 (46)	13 (100)
	HN-PGL	2 (17)	7 (58)		3 (25)	10 (83)	12 (100)
	PCC	2 (2)	3 (3)		115 (95)	118 (98)	120 (100)

Table S4. *HIF-2α-C expression in tumor samples with and without predisposing mutations*

HIF-2α-C expression		# of patients: Low intensity		# of patients: High Intensity		# of patients: Total		
Mutation	Diagnosis	Non-metastatic	Metastatic	Non-metastatic	Metastatic	Non-metastatic	Metastatic	Total
	sPGL	1	1	0	3	1	4	
*SDH*x	HN-PGL	1	0	3	0	4	0	13
	PCC	1	1	1	1	2	2	
	sPGL	-	-	-	-	-	-	
RET	HN-PGL	-	-	-	-	-	-	15
	PCC	10	0	5	0	15	0	
	sPGL	-	-	-	-	-	-	
VHL	HN-PGL	-	-	-	-	-	-	6
	PCC	5	0	1	0	6	0	
	sPGL	-	-	-	-	-	-	
NF1	HN-PGL	-	-	-	-	-	-	8
	PCC	4	0	4	0	8	0	
	sPGL	-	-	-	-	-	-	
HRAS	HN-PGL	-	-	-	-	-	-	2
	PCC	2	0	0	0	2	0	
	sPGL	1	0	0	0	1	0	
EPAS1	HN-PGL	-	-	-	-	-	-	1
	PCC	-	-	-	-	-	-	
	sPGL	0	0	4	0	4	0	
No mutation	HN-PGL	-	-	-	-	-	-	52
	PCC	31	3	14	0	45	3	
	sPGL	1	0	0	2	1	2	
Unknown	HN-PGL	-	-	8	0	8	0	47
	PCC	23	0	13	0	36	0	

Table S5. *Staining patterns for different proteins in non-metastatic vs metastatic disease.* Number and fraction (%) of cores staining with low (0-1) or high (2-3) intensity for nuclear (HIF-2α-N) or cytoplasmic (HIF-2α-C) HIF-2α as well as other proteins in non-metastatic vs. metastatic tumors.

Marker	Intensity	Low intensity cores		High intensity cores		Total # of Tumors	
	**Diagnosis**	*Non-metastatic (%)*	*Metastatic (%)*	*Non-metastatic (%)*	*Metastatic (%)*	*Non-metastatic (%)*	*Metastatic (%)*
**CgA**	*sPGL*	4 (67)	3 (50)	2 (33)	3 (50)	6 (100)	6 (100)
	HN-PGL	11 (85)	–	2 (15)	–	13 (100)	–
	PCC	10 (9)	2 (40)	105 (91)	3 (60)	115 (100)	5 (100)
**TH**	*sPGL*	3 (50)	3 (50)	3 (50)	3 (50)	6 (100)	6 (100)
	HN-PGL	13 (100)	–	0 (0)	–	13 (100)	–
	PCC	12 (11)	0 (0)	103 (89)	5 (100)	115 (100)	5 (100)
**SYP**	*sPGL*	2 (33)	1 (17)	4 (67)	5 (83)	6 (100)	6 (100)
	HN-PGL	0 (0)	–	13 (100)	–	13 (100)	–
	PCC	7 (5)	0 (0)	108 (95)	5 (100)	115 (100)	5 (100)
**VIM**	*sPGL*	1 (17)	3 (50)	5 (83)	3 (50)	6 (100)	6 (100)
	HN-PGL	0 (0)	–	13 (100)	–	13 (100)	–
	PCC	25 (22)	1 (20)	90 (78)	4 (80)	115 (100)	5 (100)
**Ki-67**	*sPGL*	0 (0)	0 (0)	7 (100)	6 (100)	7 (100)	6 (100)
	HN-PGL	0 (0)	–	13 (100)	–	13 (100)	–
	PCC	15 (13)	0 (0)	103 (87)	5 (100)	118 (100)	5 (100)
**HIF-1α**	*sPGL*	6 (100)	5 (100)	0 (0)	0 (0)	6 (100)	5 (100)
	HN-PGL	13 (100)	–	0 (0)	–	13 (100)	–
	PCC	109 (97)	4 (80)	3 (3)	1 (20)	112 (100)	5 (100)
**HIF-2α-N**	*sPGL*	7 (100)	5 (83)	0 (0)	1 (17)	7 (100)	6 (100)
	HN-PGL	8 (67)	–	4 (33)	–	12 (100)	–
	PCC	112 (99)	5 (100)	2 (1)	0 (0)	114 (100)	5 (100)
**HIF-2α-C**	*sPGL*	3 (43)	1 (17)	4 (57)	5 (83)	7 (100)	6 (100)
	HN-PGL	1 (8)	–	11 (92)	–	12 (100)	–
	PCC	76 (67)	4 (80)	38 (33)	1 (20)	114 (100)	5 (100)
**DEC1/BHLBE40**	*sPGL*	2 (33)	3 (50)	4 (67)	3 (50)	6 (100)	6 (100)
	HN-PGL	1 (8)	–	12 (92)	–	13 (100)	–
	PCC	57 (50)	3 (50)	58 (50)	2 (50)	115 (100)	5 (100)
**SDHB**	*sPGL*	3 (43)	4 (67)	4 (57)	2 (33)	7 (100)	6 (100)
	HN-PGL	2 (17)	–	10 (83)	–	12 (100)	–
	PCC	1 (1)	1 (20)	114 (99)	4 (80)	115 (100)	5 (100)

Table S6. *General and staining characteristics of the tumor samples with EPAS1 mutation.*

	Patient ID	Diagnosis	Clinical Behaviour	Survival Status	Follow-up (years)	*EPAS1* mutation	Effects of mutation on protein	Other mutations
**TMA**	#45 BS19765 A/03	sPGL	Non-metastatic	Alive at latest follow-up in 2021	18	c.1589C>T, p.Ala530Val	Increased stabilization	No
**CNIO cohort**	00T105	PCC	Non-metastatic	Unknown	16	c.1591C>T, p.Pro531Ser	Increased stabilization	No
	09T261	sPGL	Non-metastatic	Alive at latest follow-up in 2014	12	c.1592C>T, p.Pro531Leu	Increased stabilization	No
	12T286	sPGL	Non-metastatic	Alive at latest follow-up in 2012	11	c.1592C>T, p.Pro531Leu	Increased stabilization	No
	14T277	sPGL	Non-metastatic	Alive at latest follow-up in 2014	1/2	c.1591C>G, p.Pro531Ala	Increased stabilization	No

Marker	Mutation	Diagnosis	Negative (%)	Weak (%)	Intermediate (%)	Strong (%)	Positive (%)	Total # of Tumors (%)
**HIF-2α-C**	*EPAS1*	sPGL	**0 (0)**	3 (75)	1 (25)	0 (0)	**4 (100)**	4 (100)
		PCC	**0 (0)**	0 (0)	1 (100)	0 (0)	**1 (100)**	1 (100)
**HIF-2α-N**	*EPAS1*	sPGL	**1 (25)**	2 (50)	0 (0)	1 (25)	**3 (75)**	4 (100)
		PCC	**0 (0)**	0 (0)	1 (100)	0 (0)	**1 (100)**	1 (100)

Table S7. *Causes of death for PPGL patients*. Number and fraction (%) of patients dead from metastatic PPGL as compared to all causes of death.

		10-year survival			
**Cause of Death**	**Status**	**sPGL (%)**	**HN-PGL (%)**	**PCC (%)**	**Total (%)**
All	Alive	7 (54)	13 (100)	104 (85)	124 (83)
	Dead	6 (46)	0 (0)	19 (15)	25 (17)
	Total	13 (100)	13 (100)	123 (100)	149 (100)
Metastatic PPGL	Alive	7 (78)	13 (100)	104 (97)	124 (88)
	Dead	2 (22)	0 (0)	3 (3)	5 (12)
	Total	9 (100)	13 (100)	107 (100)	129 (100)
